# Clinical correlation of influenza and respiratory syncytial virus load measured by digital PCR

**DOI:** 10.1371/journal.pone.0220908

**Published:** 2019-09-03

**Authors:** Diego R. Hijano, Jessica Brazelton de Cardenas, Gabriela Maron, Cherilyn D. Garner, Jose A. Ferrolino, Ronald H. Dallas, Zhengming Gu, Randall T. Hayden

**Affiliations:** 1 Department of Infectious Diseases, St. Jude Children's Research Hospital, Memphis, TN, United States of America; 2 Department of Pathology, St. Jude Children's Research Hospital, Memphis, TN, United States of America; Hong Kong Institute for the Humanities and Social Sciences, HONG KONG

## Abstract

Acute respiratory tract infections are a major cause of respiratory morbidity and mortality in pediatric patients worldwide. However, accurate viral and immunologic markers to predict clinical outcomes of this patient population are still lacking. Droplet digital PCR assays for influenza and respiratory syncytial virus (RSV) were designed and performed in 64 respiratory samples from 23 patients with influenza virus infection and 73 samples from 19 patients with RSV infection. Samples of patients with hematologic malignancies, solid tumors, or sickle cell disease were included. Clinical information from institutional medical records was reviewed to assess disease severity. Samples from patients with fever or respiratory symptoms had a significantly higher viral loads than those from asymptomatic patients. Samples from patients with influenza virus and RSV infection collected at presentation had significantly higher viral loads than those collected from patients after completing a course of oseltamivir or ribavirin, respectively. RSV loads correlated positively with clinical symptoms in patients ≤5 years of age, whereas influenza viral loads were associated with clinical symptoms, irrespective of age. Patients receiving antivirals for influenza and RSV had a significant reduction in viral loads after completing therapy. Digital PCR offers an effective method to monitor the efficacy of antiviral treatment for respiratory tract infections in immunocompromised hosts.

## Introduction

Acute respiratory tract infections are the leading cause of morbidity and mortality in infants and children worldwide [1]. Respiratory viruses such as respiratory syncytial virus (RSV), influenza virus, parainfluenza virus, adenovirus, and picornavirus play an important role in the etiology of respiratory diseases in immunocompetent and immunocompromised patients, especially those undergoing myelosuppression and hematopoietic cell transplant [2].

Considerable advances have been made in predicting clinical outcomes for several systemic viral infections (e.g., HIV), and measuring viral load and markers of immune activation have become part of standard clinical care [3]. Unfortunately, this is not the case for influenza, RSV, and other viral respiratory infections. Despite the enormous global health impact of viral respiratory infections [4], accurate viral and immune markers to predict clinical outcomes for patients are still lacking. Several methods have been used to precisely correlate viral copy number and disease severity [5–9]. Molecular quantitative assays have been suggested to detect and monitor clinical viral respiratory disease, particularly in immunosuppressed pediatric patients [10]. However, their results are conflicting and there continues to be a lack of clinical tests to accurately measure viral load [5, 6, 8, 9]. Furthermore, respiratory infections in these patients are associated with prolonged viral shedding and concerns for emergent antiviral resistance (for those in whom the viral infection is treatable). Quantitative determination of viral load may provide an important tool to directly evaluate the efficiency of antiviral therapy, to determine if changes in therapy are necessary and to assess need for further isolation to protect transmission to other highly susceptible hosts. These problems highlight the importance and potential value of developing assays for absolute, precise and reliable quantitative viral detection to improve the accuracy of clinical decision making in immunocompromised children with viral respiratory infections.

Viral load is commonly determined by quantification of viral genomic fragments [11]. Quantitative determinations by real-time PCR are indirect and provide a relative quantification of viral load [12]. However, the precision of such assays is limited by the nature of cycle threshold (Ct) determination with resulting values having a relative standard deviation that can exceed 50% [13–15]. In addition, assay workflow and design is complicated by the need for quantitative calibrators, introducing error risk and requirements for consistent supplies of well-characterized, commutable reference material. The latter can be particularly difficult to obtain for less commonly quantified viruses in general, and particularly for RNA viruses, such as those studied here [16–21]. Marked variation in assay performance characteristics and in materials used as calibration has been described. The implementation of international standards, such as those that have been made available by the World Health Organization (WHO) has led to reduction of this problem. However, standards are only available for a limited number of pathogens and can vary over time [22, 23].

Digital PCR (dPCR) is a PCR method for the detection and quantitation of nucleic acids [14, 18, 24]. dPCR offers several advantages over real time methods [21, 25]. Digital PCR differs from real-time methods in the way the sample target is measured, by dividing up the reaction into multiple, smaller endpoint reactions which allows direct absolute quantification of the target [17, 26, 27]. dPCR methods do not depend on calibration curves [19, 25, 28] and are less subject to enzyme inhibition as they employ endpoint amplification [26, 29, 30]. They are more robust in the presence of viral sequence heterogeneity, showing less susceptibility to mismatches between primers-probe patient viral sequence. Such miss-matches are more likely to cause false-negative results or under-quantitation using qPRC, compared to dPCR [11, 31, 32]. This is particularly important for RNA viruses which have high mutation rates and need only a few weeks to escape immune response or to produce drug-resistant mutants [31, 33–36]. Finally, clinical results generated by dPCR are more precise, reliable and reproducible than those by real-time PCR, thereby highlighting its potential diagnostic, prognostic, and predictive utility. Although dPCR has been previously used for quantitative detection of systemic viruses [17, 37, 38], its use has been more limited for localized viral infections such as those in the respiratory tract.

In this study, we analytically and clinically validate two dPCR assays for quantitative detection of influenza virus and RSV in pediatric patients with hematologic malignancies, solid tumors, and Sickle-cell disease (SCD). Viral loads are also correlated with clinical symptoms and provide one of the first demonstrations of this technology for monitoring therapeutic response to antiviral agents.

## Materials and methods

### Ethical approval

The study was approved by St. Jude institutional review board. All samples were fully anonymized before initiation of the study and the IRB committee waived the requirement for informed consent.

### Study population

St. Jude is a quaternary referral center for pediatric patients with cancer, including those requiring hematopoietic cell transplant (HCT) and patients with SCD. Pediatric patients with hematologic malignancies, solid tumors, or SCD with 2 or more consecutive respiratory samples positive for influenza virus or RSV were retrospectively identified through results of routine clinical testing by qualitative PCR (qPCR; FilmArray, BioFire Diagnostics) at St. Jude Children’s Research Hospital (St. Jude, Memphis, TN) from 2014 to 2017. Medical records were reviewed to extract information on age, gender, race, primary diagnosis, date of infection with influenza or RSV, symptoms and clinical course, other laboratory results, supportive and antiviral treatment. Lower respiratory tract infection (LRTI) was defined by crackles, hypoxia, tachypnea, or apnea and/or radiographic evidence of pneumonia (infiltrates or consolidation). The institutional infection control and prevention policies mandate that patients with either influenza or RSV infection have a negative test in order to discontinue isolation precautions. As a result, many patients get subsequent testing even in the absence of symptoms. Patients were considered asymptomatic if there were no symptoms at the time of sample collection. This study was approved by the St. Jude institutional review board.

### Digital PCR assay

Remnant samples from nasopharyngeal washes obtained from 2014 to 2017 for routine care were used for this study. Washes were performed using normal saline and immediately transported to the Clinical Virology Laboratory on ice and stored at 4°C until testing (within 24 hours of collection). Sample remaining after clinical testing was stored for quality assurances at –80°C until used in this evaluation. Digital PCR (dPCR) assays for influenza were performed using the RainDrop digital PCR system (RainDance Technologies) and ViroReal assay kits (Ingenetix). Viral RNA stock generated by *in vitro* synthesis and purchased from Ingenetix was used for analytical validation of the assay, along with viral stock material generated by ATCC (quantified by ATCC using dPCR, plaque-forming units, and 50% tissue culture infective dose) and Zeptometrix. The assay for RSV was also designed for the RainDrop digital PCR system and primer/probes previously designed for qPCR [39]. Synthetic viral RNA stock for analytical validation of the RSV assay was purchased from Bio-Synthesis, Inc., along with additional viral stock material from ATCC and Zeptometrix.

After thawing at room temperature, nucleic acids were purified from 200 μL each of the previously frozen samples using the Specific A protocol of the bioMérieux NUCLISENS easyMAG nucleic acid extractor and stored at –20°C. An internal control, intype IC-RNA (QIAGEN, Inc), was added to and extracted with each sample and read using the VIC channel during dPCR. dPCR was conducted using the RainDrop Digital PCR system. ViroReal assay kits (Ingenetix) were used for influenza A and B assays, whereas RSV assay primer/probes were adapted from a previously designed, laboratory-developed qRT-PCR assay [39]. An assay volume of 25 μL was used for all virus samples. Standard qPCR protocols from Ingenetix and QuantaBio were used for the master mix and cycling conditions. After cycling, reactions were transferred to the RainDrop reader and data were analyzed using RainDrop Analyst II software (v1.0.0.520). Results of copies per reaction were converted to log_10_ copies per milliliter of the original patient sample pre-extraction.

### Study validation and design

Validations for influenza and RSV included multiple lot numbers of all reagents and spanned several months allowing between run variability to be assessed. All samples had Qiagen IPC added prior to extraction to control for inhibition and ranged from 5,000–15,000 copies/mL. Cross reactivity for all three assays was tested using the Zeptometrix respiratory panel. Validation also included determination of limit of detection, positive cut-off value, linearity, within and between run variability, and limit of quantitation. The limit of blank (LOB) was defined by the 95% cutoff for all no template controls and was considered a baseline value to eliminate false positive results due background noise. LOD_95_ was initially calculated by determining the lowest non-zero concentration for which 95% of the replicates gave a positive result. The LOQ was based on the percent CV for linearity runs (lowest level with a CV <10%). As calculated LOD_95_ and LOQ were both lower than the LOB. Clinical samples used in the validation were run blinded.

### Statistical analyses

**Fig 1:**
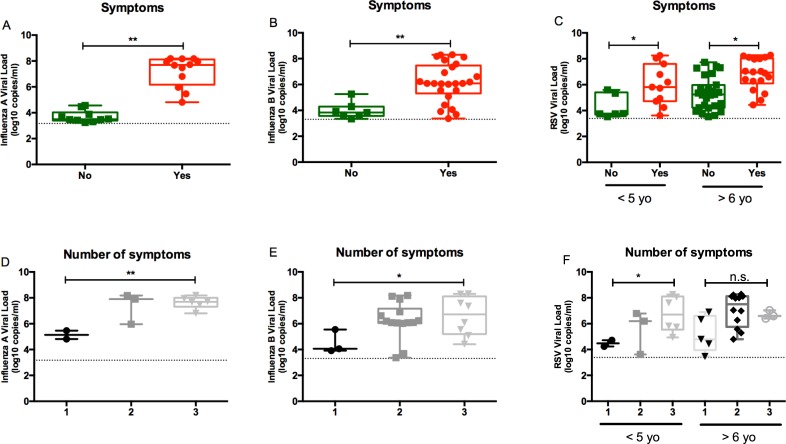
Correlation of influenza virus and RSV viral loads with clinical infection. Viral load and presence of fever, cough, and/or nasal congestion for (A) influenza virus A, (B) influenza virus B, and (C) RSV. Viral load and presence of 1–3 symptoms for patients with (D) influenza A, (E) influenza B, and (F) RSV infection. *P* values from mixed linear regression model *P < 0.05; **P < 0.01; ***P < 0.001. yo, years old; viral loads (log_10_ copies/ml). Dotted line represents lower limit of quantification for each assay.

**Fig 2:**
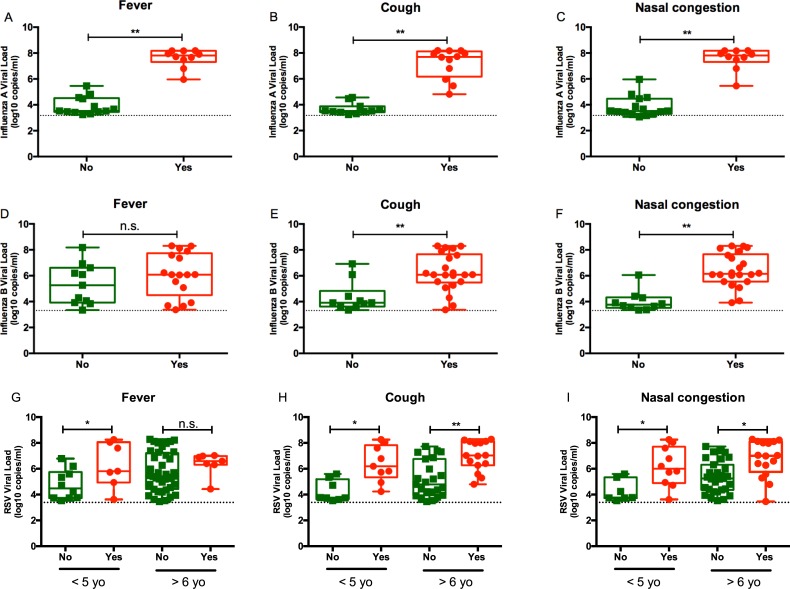
Correlation of influenza viral load with clinical signs/symptoms. Influenza A viral load and presence of (A) fever, (B) cough, and/or (C) nasal congestion. Influenza B viral load and presence of (D) fever, (E) cough, and/or (F) nasal congestion. RSV viral load and presence of (G) fever, (H) cough, and/or (I) nasal congestion. *P* values from mixed linear regression model *P < 0.05; **P < 0.01; ***P < 0.001. Viral loads (log_10_ copies/ml). Dotted line represents lower limit of quantification for each assay.

**Fig 3:**
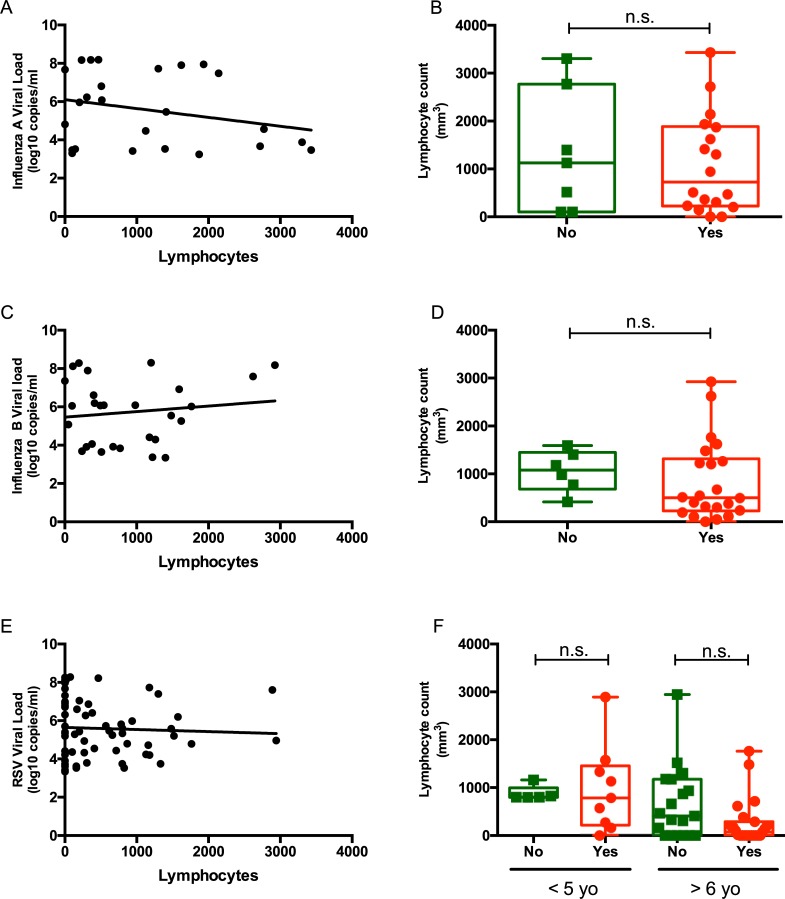
Correlation of lymphopenia with viral load and clinical symptoms. Correlation between ALC and (A) influenza A viral load, (C) influenza B viral load, and (E) RSV viral load (E). ALCs and the presence of clinical symptoms for (B) influenza A, (D) influenza B, and (F) RSV. *P* values from mixed linear regression model *P < 0.05; **P < 0.01; ***P < 0.001. Viral loads (log10 copies/ml).

Descriptive statistical analyses are presented as proportions for categorical data and mean ± standard deviation for continuous data. Statistical analysis was performed using the chi-squared test or Fisher’s exact test, as appropriate, for categorical data, and Student’s t-test for continuous variables. To account for difference in the number of samples per patient, a mixed linear regression model was used to determine association between viral load and clinical characteristics [Presence of symptoms, Number of symptoms and individual symptoms: Fever, Cough and Nasal Congestion) (Figs 1 and 2), Lymphocyte count (Fig 3), Presence/absence of co-infections, and Number of additional viruses (S2 Fig)], with the random variable being the patient identifier. A similar mixed linear regression model approach was used to determine association between lymphocyte count and presence of symptoms. Logistic regression using initial viral load upon clinical presentation was used to assess the correlation between viral load and clinical complications in univariate and multivariate analysis. Paired t-tests was used to compare samples before and after treatment. A P-value < 0.05 was considered statistically significant.

## Results

### Performance characteristics of dPCR

Analytic specificity for influenza A were 100%. Positive predictive value was 0.793 and negative predictive value was 0.818 (S1 Table). Plotted linearity for influenza A had an R^2^ value of 0.997 (S1 Fig). Within run variation ranged from 0.24% CV to 0.85% CV for the high external control, and from 0.42% CV to 0.64% CV for the low external control. Between run variation was 0.87% CV for the high control and 0.70% CV for the low control (S2 Table). For influenza A the limit of detection, limit of quantitation, and cutoff for positivity all coincided at 3.172 log_10_ copies/mL (S1 Table).

Analytic specificity was 100% for influenza B. The positive predictive value was 1 and the negative predictive value was 0.682 (S1 Table). Plotted linearity for influenza B had an R^2^ value of 0.9887 (S1 Fig). Within run variation ranged from 0.06% CV to 0.56% CV for the high control, and 0.49% to 8.37% CV for the low control. Between run variation was 0.35% CV for the high control and 4.8% CV for the low control (S3 Table). The limit of detection, limit of quantitation, and cutoff for positivity coincided at 3.309 log_10_ copies/mL.

Analytic specificity for RSV was 100%. The positive predictive value was 0.969 and negative predictive value was 0.565 (S1 Table). Plotted linearity for RSVA had an R^2^ value of 0.96, while plotted linearity for RSVB had an R^2^ value of 0.93 (S1 Fig). The within run variation ranged from 0.05% CV to 0.55% CV for the high control and 0.56% to 0.94% for the low control. The between run variation was 0.31% CV for the high control and 0.86% CV for the low control (S4 Table). The limit of detection, limit of quantitation, and cutoff for positivity coincided at 3.389 log_10_ copies/mL. No cross-reactivity was detected for any of the three assays.

### Demographic and clinical characteristics

The analysis included 137 samples from 42 patients (64 samples from 23 patients with influenza virus infection and 73 samples from 19 patients with RSV infection). Table 1 shows demographic and clinical characteristics of patients. Five patients had a coinfection with influenza virus and RSV. Median number of samples per patient was 2 (range 2–6) for influenza and 3 (range 2–10) for RSV. Median age was 6 years (range 2–19) for patients with influenza virus infection and 8 years (range 1–21) for those with RSV infection. Ten patients with influenza virus infection and six patients with RSV infection were ≤5 years of age. Of the 42 patients, 21 (50%) were male. By race, 20 (47.62%) patients were white, 19 (45.24%) were African American, and 3 (7.14%) were Hispanic. By diagnosis, 25 (59.52%) patients had leukemia, 10 (23.80%) had solid tumors, 6 (14.28%) had SCD, and 1 (2.38%) had hemophagocytic lymphohistiocytosis. By virus type and strain, 13 (30.95%) patients had influenza B, 9 (21.43%) had influenza A H3, 1 (2.38%) had influenza A H1N1, and 19 had RSV (45.24%). Eighteen (42.86%) patients had chemotherapy delayed due to the viral infection (3 with Influenza A; 7 with Influenza B and 8 with RSV). Of 5 patients having coinfection of influenza and RSV, influenza infection was detected first in 2 patients (in the series of samples from each patient), RSV was detected first in 1 patient, and both viruses were detected simultaneously in the initial positive sample in 2 patients. Two patients had influenza A and influenza B coinfection. Median interval between collected samples was 10 days (range 1–49 days). Of 137 samples initially positive in BioFire FilmArray^®^ analysis, 19 (13.86%) were below the established limit of quantification by dPCR and were considered negative: 2 (8.0%) for influenza A, 7 (17.95%) for influenza B, and 10 (13.69%) for RSV. All these corresponded to samples obtained after antiviral treatment was completed. For samples included in the validation, the specificity for influenza A was 82.05%, influenza B, 100%, and RSV was 86.66%. The clinical sensitivity was 92% for influenza A, 82.05% for influenza B, and 86.30% for RSV.

**Table 1:** Demographic and clinical characteristics of patients (*n* = 42).

Variables	Respiratory viral infections (n = 42)			*P* value
	Influenza A (*n* = 10)	Influenza B (*n* = 13)	RSV (*n* = 19)	
**Gender (male)**	5 (23.81%)	7 (33.3%)	9 (42.86%)	1
**Age** (mean [**range**], **years**)	8.73 (3.71–18.17)	7.83 (1.71–18.65)	9.70 (1.34–21.31)	0.84
**Race**				
White	5 (25%)	5 (25%)	10 (50%)	0.27
African American	5 (26.32%)	8 (42.11%)	6 (31.58%)	
Hispanic	0	0	3 (100%)	
**Diagnosis**				
Leukemia	2 (8%)	10 (40%)	13 (52%)	0.02
Solid tumor	4 (40%)	2 (20%)	4 (40%)	
Sickle cell disease	4 (66.67%)	1 (16.67%)	1 (16.67%)	
HLH	0	0	1(100%)	
**Initial symptoms**				
LRTI^a^	0	7 (53.85%)	6 (46.15%)	0.02
Oxygen	0	3 (27.27%)	8 (72.73%	0.03
ICU	0	2 (40%)	3 (60%)	0.49
Coinfection	6 (25%)	8 (33.33%)	10 (41.67%)	0.84
Absolute lymphocyte count	986 (0–2142)	958 (49–2925)	775.4 (0–2944)	0.80
Antiviral therapy^b^	10 (100%)	13 (100%)	8 (42.11%)	<0.001
Death	0	0	2 (10.53%)	0.49

^a^LRTI characterized by crackles, hypoxia, tachypnea, or apnea and/or radiographic evidence of pneumonia (infiltrates or consolidation).

^b^Oseltamivir for influenza and ribavirin for RSV

Abbreviations: HLH, hemophagocytic lymphohistiocytosis; ICU, intensive care unit; LRTI, lower respiratory tract infection; RLS, respiratory syncytial virus.

### Correlation between respiratory signs/symptoms and viral load

Viral loads were significantly higher in patients with fever, cough, or nasal congestion than in patients asymptomatic for influenza A (*P* < 0.001), influenza B (*P* < 0.001), and RSV (*P* < 0.001) (Fig 1A–1C) infection. Viral loads in patients with all 3 signs/symptoms were higher than for those having only 1 sign/symptom (Fig 1D–1F). These differences were significant for patients with influenza A (*P* < 0.01), influenza B (*P* < 0.05), and RSV (*P* < 0.01) infections.

Fever was associated with higher viral load only in patients with influenza A infection (*P* < 0.001) and not in those with influenza B infection (Fig 2A and 2D). Viral load for patients with RSV was significantly higher in febrile patients ≤5 years of age (*P* < 0.05), but this difference was not significant for older patients (Fig 2G). Patients with cough had significantly higher viral loads than those who did not have these signs/symptoms, irrespective of age or virus type (Fig 2B–2E–2H). While nasal congestion was significantly associated with higher viral for Influenza A and Influenza B, only patients > 5 years of age with nasal congestion and RSV infection had significantly higher viral load when compared to those who did not report this symptom (Fig 2C–2F–2I).

### Coinfection

Twenty-two patients had coinfection with 1 or more respiratory viruses. There was no difference in influenza virus or RSV viral loads (S2 Fig) between patients with single infection and those with coinfections. This result was irrespective of the number of viruses detected (S2 Fig). The most common viruses associated with influenza or RSV were rhinovirus or enterovirus, followed by coronavirus OC43 and parainfluenza type 3. Oxygen requirement, intensive care unit (ICU) admission, and number of deaths did not differ between patients with single infection and those with coinfections.

### Absolute lymphocyte count

Twenty-six (61.90%) of patients had an absolute lymphocyte count less than 1000/mm^3^ at the time of diagnosis. Fourteen (33.33%) had an ALC less than 300mm^3^, with 8 of them having severe lymphopenia (ALC <100). Viral load was not significantly correlated with ALC for patients with influenza or RSV infection (Fig 3A, 3C and 3E), regardless of age. ALC was also not associated with the presence of clinical symptoms in patients with influenza A, influenza B, or RSV infection (Fig 3B, 3D and 3F).

### Lower respiratory tract disease

Lower respiratory tract infection (LRTI) developed in 13 (30.95%) patients: 7 (53.85%) had influenza B and 6 (46.16%) had RSV infections. Only 11 (26.9%) patients required oxygen during admission: 3 had influenza B and 8 had RSV infection. Further, 5 patients, 2 with influenza B and 3 with RSV infection, were admitted to the ICU. Initial viral load was not significantly higher in these patient subgroups when compared to those that did not have clinical complications (Table 2). Two patients that died in relation to RSV infection had an ALC of 0, had developed LRTI and were in the ICU at the time of death. Univariate and multivariate analyses including age, initial viral load, and ALC as covariates did not reveal significant associations with clinical complications (Table 3). In addition, initial viral load and initial ALC were not significantly associated when considering all these complications together (S2 Fig).

**Table 2:** RSV and influenza B initial viral loads and disease severity.

Variable	RSV (mean, 95% CI)	OR (95% CI)	*P* value	Influenza B (mean, 95% CI)	OR (95% CI)	*P* value
**LRTI**^**a**^						
Yes	6.66 (4.6–8.72)	1.09 (0.61–1.95)	0.76	6.25 (4.450–8.01)	1.24 (.69–2.21)	0.46
No	6.4 (5.34–7.46)	Reference		5.44 (3.05–7.83)	Reference	
**Oxygen**						
Yes	7.33 (6.46–8.20)	1.88 (.89–3.94)	0.09	5.72 (1.14–12.59)	.95 (0.49–1.84)	0.88
No	5.87 (4.54–7.19)	Reference		5.92 (4.52–7.32)	Reference	
**ICU**						
Yes	7.57 (6.15–8.99)	1.96(0.58–6.68)	0.28	7.18 (7.13–21.49)	1.64 (0.59–4.55)	0.34
No	6.28 (5.29–7.27)	Reference		5.64 (4.24–7.03)	Reference	
**Death**						
Yes	7.55 (0.32–14.79)	1.86(.44–7.79)	0.39	N/A	N/A	N/A
No	6.36 (5.42–7.29)	Reference		Reference	Reference	

^a^Lower respiratory tract infection is characterized by crackles, hypoxia, tachypnea, or apnea and/or radiographic evidence of pneumonia (infiltrates or consolidation).

Abbreviations: CI, confidence interval; ICU, intensive care unit; N/A, not applicable; OR, odds ratio; RSV, respiratory syncytial virus

**Table 3:** Univariate and multivariate analysis for complications related to viral respiratory infections.

Variable	Univariate analysis		Multivariate analysis	
	OR (95% CI)	*P* value	Odd ratio (CI 95%)	*P* value
Age	0.98 (0.87–1.11)	0.8	0.97 (0.85–1.10)	0.63
Viral load^a^	0.94 (0.59–1.49)	0.79	0.95 (0.58–1.57)	0.84
ALC^b^	1 (0.99–1.01)	0.67	1 (0.99–1.01)	0.59

^a^Initial viral load during clinical presentation (log10 copies/ml).

^b^Initial ALC upon clinical presentation.

Abbreviations: ALC, absolute leukocyte count; CI, confidence interval.

### Treatment

**Fig 4:**
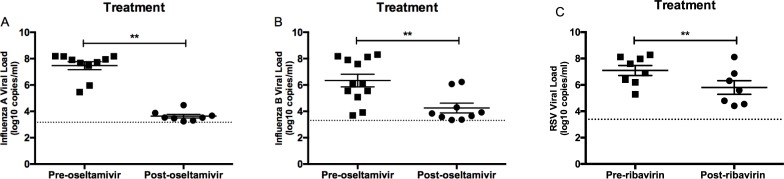
Influenza and RSV viral loads after antiviral treatment. Influenza viral load before and after oseltamivir treatment for (A) influenza A and (B) influenza B. (C) RSV viral load before and after ribavirin treatment. *P < 0.05; **P < 0.01; ***P < 0.001. Viral loads (log_10_ copies/ml); Dotted line represents lower limit of quantification for each assay.

All patients with influenza infection were treated with oseltamivir within 24 h of diagnosis. Influenza viral load after receiving treatment was significantly lower for both patients with influenza A (*P* < 0.01) and influenza B (*P* < 0.01) infections (Fig 4A and 4B). Ribavirin was administered to 8 (42.1%) patients with RSV infection: 7 patients inhaled ribavirin, and 1 patient received intravenous ribavirin since the patient was on mechanical ventilation (Table 1). Viral load was significantly reduced (*P* < 0.01; Fig 4C) after completion of treatment.

## Discussion

We developed and validated two dPCR assays for quantitative detection of influenza virus (64 samples from 23 patients) and RSV (73 samples from 19 patients) in respiratory tract samples from patients with hematologic malignancies, solid tumors, and SCD. Both in patients with influenza virus and RSV infection, viral loads were associated with symptoms of infection. Furthermore, patients receiving antiviral treatment had a significantly decreased viral load after completion of therapy.

The correlation between viral load and severity of infection remains controversial for respiratory viruses [6, 8, 9, 15, 40]. For RSV, viral load was significantly associated with severity or presence of respiratory illness in children ≤5 years of age. An analysis of cycle threshold values and disease severity in 4410 patients by Fuller et al. showed that in those with RSV infection, viral load was associated with disease severity only in patients younger than 2 years of age [15]. DeVincenzo et al. reported that patients intubated because of RSV-induced respiratory distress had higher viral loads than those who were not, and that higher viral loads were associated with longer hospitalization [5]. We could not measure viral loads in lower respiratory tract samples in our study. However, a study showed that viral loads in the upper and lower respiratory tracts are strongly correlated when measured at the same time [41]. In an autopsy study of patients with fatal RSV LTRI, the viral antigen was extensively present in the lung tissue [42]. This points to a possible association between viral load and RSV disease severity. However, many studies have not found this association [6, 7]. Gerna et al. reported a direct relationship between viral loads and clinical symptoms in their patient cohort, but viral loads were not associated with an increased risk of developing bronchiolitis [7].

Findings from previous studies on the association between influenza viral load and disease severity, as in the case of RSV, are inconsistent. Some studies report higher viral loads in those with more severe viral disease [9, 43, 44], whereas others do not show this association [6, 8, 45, 46]. Fuller et al. reported that patients hospitalized for influenza infection had higher viral loads than those managed on an outpatient basis and asymptomatic controls [15]. Lu et al. described a significant association between higher H1N1 influenza virus load and pneumonia [44]. In contrast, Meschi et al. found no significant increase in mean values of viral RNA concentration in patients with pneumonia. This inconsistency in results may be due to lack of standardized methods to quantify respiratory viruses in respiratory secretions, technical difficulties in precisely quantifying viral load at the mucosal surface, and the need to collect samples over several days for accurate analysis.

Studies on viral dynamics in coinfections and its impact on disease severity have recently gained interest, but results have been inconsistent. Some studies show that viral coinfections are similar to single-virus infections [47, 48], whereas others report an association between viral coinfection and increased [49, 50] or decreased [51, 52] disease severity. Our study did not find significant differences between viral load and signs/symptoms in patients with viral coinfections versus those with a single infection. The presence of one or more viruses did not impact RSV or influenza viral load. Pinky et al. showed that fast-growing viruses can impair the replication of remaining viruses during a coinfection, whereas slow-growing viruses can be inhibited in the presence of other viruses [53]. This mechanism of viral competition during coinfections might explain the discrepancies among studies [53].

Another study showed that the relationship between viral loads and multiple virus infections is virus specific [52]. The limited sample size of our study did not allow us to further explore viral load in relation to each different virus, which might explain why we did not see an overall change in influenza or RSV viral loads in cases of viral coinfection.

Lymphocytes play an important role in viral respiratory infection [42, 54]. An ALC count below 100/mm^3^ or 300/mm^3^ is significantly associated with increased risk of progression to LRTI [42, 55]. In addition, patients receiving T-cell therapy have decreased viral loads, viral clearance, and resolution of clinical symptoms [56]. Although symptomatic patients had a lower lymphocyte count than did asymptomatic patients, this difference was not significant by age or virus.

The association between viral load and therapeutic response seen in our study has some precedents in the literature [57, 58]. Reduction of influenza and RSV viral loads by using different treatments has been reported in several animal models [59, 60]. However, few studies report the reduction of viral loads after treatment that are associated with clinical improvement in humans, particularly children at high risk of severe infection. Clinical trials of adults experimentally infected RSV show reduction in viral loads after treatment [61, 62]. Khanna et al. reported a decrease of >2 log_10_ copies/mL in RSV load in 11 (58%) of 19 adult patients with hematologic malignancies within 7 days of initiating treatment [63]. Several case reports and case series have described the role of oseltamivir in decreasing viral load in respiratory secretions [40, 64, 65]. The significant reduction in viral load for both influenza virus and RSV seen in our study agrees with these studies.

Our study is relevant because no studies have been previously published on the use of dPCR to determine viral loads of RSV and influenza virus in pediatric patients with hematologic malignancies, solid tumors, or SCD. Assays reported to date have been performed using real-time PCR. dPCR provides advantages such as endpoint quantitation without the need for calibration curves, improved precision, and reduced likelihood of quantitative bias based on amplification efficiency or inhibition [17, 26, 27]. From 137 samples that were initially positive by BioFire FilmArray®, 19 were negative by dPCR. All 19 samples were collected after completion of antiviral treatment. This might be anticipated due to subsampling error in patients with very low viral load or due to some degree of sample degradation in similarly low load samples (near the assay LOD). These results are in agreement with previous work from our group and others on cytomegalovirus, showing that although dPCR exhibits increased precision over qPCR at higher viral loads, diminished sensitivity can be seen in samples with low viral load values, potentially attributable to reduced input sample volume in the dPCR system [66–68].

Our study was limited by its sample size. Patients were selected on the basis of availability of samples that previously tested positive for either influenza virus or RSV. Hence, the timing of acquiring the second or subsequent samples with respect to clinical disease and treatment varied among patients, as did the volume retrieved during nasal washes collection. Clinical information was obtained retrospectively by chart review. Given the lack of commercially available quantitative standards it is difficult to develop quantitative real time PCR methods. Therefore, we did not develop a real time assay to perform a direct comparison of quantification between real-time methods and dPCR but rather sought to develop and validate a digital PCR assay that performs well, correlates with clinical disease and can be used to monitor response to antiviral therapy. A highlight of our study was that most patients in our cohort were immunocompromised and represented a population in which respiratory infections can be severe and could therefore potentially benefit from reliable measurement of viral loads.

## Conclusion

We found that RSV viral load correlates with the presence of clinical symptoms in patients ≤5 years of age, whereas influenza viral load is associated with presence of clinical symptoms, irrespective of age. Patients receiving antiviral agents had a significant reduction in viral loads of both influenza virus and RSV after completion of therapy. Digital PCR offers an effective method to monitor the efficacy of antiviral treatment for respiratory tract infections in immunocompromised hosts with the potential to aid in decisions regarding therapy and isolation. Large-scale prospective studies are needed to explore the potential benefits of dPCR-based methods for risk stratification and their impact on patient management.

## Supporting information

S1 Fig Linearity plot during validation. Plot and linearity of measured concentrations (log10 copies/ml) from original sample and known control for (A) Influenza A, (B) Influenza B, (C) RSV A, and (D) RSV B.
(EPS)

S2 Fig Influenza and RSV viral load does not show significant differences in the presence of viral coinfections when compared to single virus infections. Viral load (log10 copies/ml) in single infections and coinfections for (A) influenza A, (B) influenza B and (C) RSV. Viral load in the presence of 1, 2, and 3 or more viruses for (D) influenza A, (E) influenza B and (F) RSV. *P* values from mixed linear regression model *P < 0.05; **P < 0.01; ***P < 0.001. Dotted line represents lower limit of quantification for each assay.
(EPS)

S3 Fig Complications of respiratory viral infections are not associated with viral load or lymphocyte count. Initial viral load (log10 copies/ml) and the presence of clinical complications for influenza B (A) and RSV (C). Initial absolute lymphocyte count and the presence of clinical complications for influenza B (B) and RSV (D). *P < 0.05; **P < 0.01; ***P < 0.001. Dotted line represents lower limit of quantification for each assay.
(EPS)

S1 File Institutional policy for collection of nasopharyngeal-washes. (PDF)

S1 Table Validation summary for respiratory assays. (DOCX)

S2 Table Influenza A precision analysis. (DOCX)

S3 Table Influenza B precision analysis. (DOCX)

S4 Table RSV AB precision analysis. (DOCX)
