# Supplementary material for: Clinical correlation of influenza and respiratory syncytial virus load measured by digital PCR
Source: PLoS One. 2019 Sep 3;14(9):e0220908. doi: 10.1371/journal.pone.0220908 (PMC6720028; doi:10.1371/journal.pone.0220908)
Supplement: S1 File — (PDF) [file pone.0220908.s004.pdf]

St. Jude Children's Research Hospital
ALSAC • Danny Thomas, Founder
Finding cures. Saving children.

NURSING POLICY & PROCEDURE MANUAL VOLUME II

Date of Original: 11/2003

Date of Review: 10/2012, 03/2014

Date of Revision: 03/2006, 07/2007,03/2014, 05/2018

NASOPHARYNGEAL WASH

PURPOSE: To provide guidelines for effective collection of nasopharyngeal wash specimens from pediatric patients, including immunocompromised patients.

POLICY: Nasopharyngeal wash specimen collection requires a physician order and may be obtained by an RN, LPN, RT, NP, PA or MD.

EQUIPMENT:

1. Nasopharyngeal aspiration kit
2. Gloves, non-sterile
3. Face shield
4. Specimen collection bag with ice
5. Appropriate computer-generated specimen label
6. Red cap

PROCEDURE:

1. Confirm Positive Patient Identification (PPID)
2. Explain the procedure to the parent(s) consistent with expressed preferred learning style and to the patient according to age and developmental level.
3. Make appropriate referrals: Child Life, Rehab, Nutrition, etc. according to assessed patient need.
4. Wash hands with antimicrobial soap and dry well or use an alcohol based product.
5. Specimen should be obtained immediately when order is written.
6. Apply non-sterile gloves and face shield. Don appropriate Personal Protective Equipment (PPE) which may include gown, gloves and face shield. Since small particle aerosols can be generated during this procedure, protection of the eyes, nose and mouth by wearing a face shield and mask is recommended
7. Place patient supine with neck extended.

8. Remove syringe cap and attach luer catheter to syringe.

9. Measure volume of saline according to the following guidelines:

   - ≤ 12 months use 1 ml
   - 13 months to 35 months use 2 ml
   - ≥ 36 months use 3 ml
   - expel excess saline

10. Apply water soluble lubricant to tip of catheter.

11. Pull back approximately 0.5mL air into the syringe prior to inserting the catheter.

12. Instruct patient to hold their breath if possible.

13. Advance catheter along the floor of the nose to the age-appropriate mark on tube or until resistance is met.

14. Quickly instill saline and immediately pull back sample into syringe.

15. Detach the catheter and apply a red cap to the syringe.

16. Label syringe according to hospital policy & procedure and place specimen in a sealed plastic bag without ice. Place sealed bag in a separate bag with ice and send to lab.

Note:

1. Nasal wash may be contraindicated in patients with the following conditions: severe thrombocytopenia with clinical symptoms, epistaxis, nasal lesions or trauma to nares, or respiratory distress (nasal flaring, retractions). Discuss safety and appropriateness of obtaining a nasal wash in patients with any one of these conditions with the patient's clinician prior to specimen collection.

2. Potential side effects may include: tickling sensation of nose and or throat, salty taste in the back of the throat, watering eyes, bleeding (due to trauma of the nasal mucosa), coughing, gagging or aspiration.

REFERENCES:

National Jewish Medical and Research Center. (2012). Nasal Wash Treatment Retrieved October 5, 2012, from http://www.njc.org/disease-info/treatments/alt-ther/nasal-wash.aspx

Npak nasopharyngeal aspiration. (2009). Retrieved October 5, 2012 from http://www.n-pak.com/
