# Supplementary material for: Clinical correlation of influenza and respiratory syncytial virus load measured by digital PCR
Source: PLoS One. 2019 Sep 3;14(9):e0220908. doi: 10.1371/journal.pone.0220908 (PMC6720028; doi:10.1371/journal.pone.0220908)
Supplement: S1 Table — (DOCX) [file pone.0220908.s005.docx]

Supplementary Table 1: Validation summary for respiratory assays.

|  | **Influenza A** | **Influenza B** | **RSV** |
| --- | --- | --- | --- |
| **Cutoff for Positivity** | 3.172 | 3.309 | 3.389 |
| **Total Tested Positives** | 25 | 39 | 73 |
| **Total Tested Negatives** | 15 | 15 | 15 |
| **False Positives** | 6 | 0 | 2 |
| **False Negatives** | 2 | 7 | 10 |
| **True Positives** | 23 | 32 | 63 |
| **True Negatives** | 9 | 15 | 13 |
| **Positive Predictive Value** | **0.793** | **1.000** | **0.969** |
| **Negative Predictive Value** | **0.818** | **0.682** | **0.565** |
| **Clinical Sensitivity** | **92.000** | **82.051** | **86.301** |
| **Clinical Specificity** | **82.051** | **100.000** | **86.667** |

^ Values for false positives and negatives were determined by the cutoffs for

positivity, shown here in log10 copy/mL original sample.
