# Supplementary material for: Clinical correlation of influenza and respiratory syncytial virus load measured by digital PCR
Source: PLoS One. 2019 Sep 3;14(9):e0220908. doi: 10.1371/journal.pone.0220908 (PMC6720028; doi:10.1371/journal.pone.0220908)
Supplement: S2 Table — (DOCX) [file pone.0220908.s006.docx]

Supplementary Table 2: Influenza A precision analysis

| **Within Run Variation** | | | | | | |
| --- | --- | --- | --- | --- | --- | --- |
|  | **Log6.6 Control** | | | **Log4.6 Control** | | |
|  | **Mean** | **St. Dev.** | **%CV** | **Mean** | **St. Dev.** | **%CV** |
| **Run 1** | 6.672537 | 0.056955 | 0.853572 | 4.510115 | 0.029001 | 0.643016 |
| **Run 2** | 6.583104 | 0.019669 | 0.298779 | 4.562865 | 0.019215 | 0.421128 |
| **Run 3** | 6.585412 | 0.015817 | 0.240189 | 4.538823 | 0.028796 | 0.634432 |
| **Between Run Variation** | | | | | | |
|  | **Log6.6 Control** | | | **Log4.6 Control** | | |
|  | **Mean** | **St. Dev.** | **%CV** | **Mean** | **St. Dev.** | **%CV** |
|  | 6.61957 | 0.057462 | 0.86807 | 4.539736 | 0.031706 | 0.698413 |
