# Supplementary material for: Clinical correlation of influenza and respiratory syncytial virus load measured by digital PCR
Source: PLoS One. 2019 Sep 3;14(9):e0220908. doi: 10.1371/journal.pone.0220908 (PMC6720028; doi:10.1371/journal.pone.0220908)
Supplement: S3 Table — (DOCX) [file pone.0220908.s007.docx]

Supplementary Table 3: Influenza B precision analysis

| **Within Run Variation** | | | | | | |
| --- | --- | --- | --- | --- | --- | --- |
|  | **Log6.6 Control** | | | **Log4.6 Control** | | |
|  | **Mean** | **St. Dev.** | **%CV** | **Mean** | **St. Dev.** | **%CV** |
| **Run 1** | 6.638459843 | 0.037201129 | 0.560387947 | 4.599125 | 0.022677803 | 0.49309 |
| **Run 2** | 6.625048141 | 0.030675746 | 0.463026758 | 4.591204 | 0.023387034 | 0.509388 |
| **Run 3** | 6.650466008 | 0.005453148 | 0.081996478 | 4.679804 | 0.029901876 | 0.638956 |
| **Run 4** | 6.645743847 | 0.00401744 | 0.060451315 | 4.922514 | 0.412194123 | 8.37365 |
| **Between Run Variation** | | | | | | |
|  | **Log6.6 Control** | | | **Log4.6 Control** | | |
|  | **Mean** | **St. Dev.** | **%CV** | **Mean** | **St. Dev.** | **%CV** |
|  | 6.63992946 | 0.023055116 | 0.347219293 | 4.698162 | 0.22552501 | 4.800282 |
