# Supplementary material for: Clinical correlation of influenza and respiratory syncytial virus load measured by digital PCR
Source: PLoS One. 2019 Sep 3;14(9):e0220908. doi: 10.1371/journal.pone.0220908 (PMC6720028; doi:10.1371/journal.pone.0220908)
Supplement: S4 Table — (DOCX) [file pone.0220908.s008.docx]

Supplementary Table 4: RSV AB precision analysis

| **Within Run Variation** | | | | | | |
| --- | --- | --- | --- | --- | --- | --- |
|  | **Log6.6 Control** | | | **Log4.6 Control** | | |
|  | **Mean** | **St. Dev.** | **%CV** | **Mean** | **St. Dev.** | **%CV** |
| **Run 1** | 6.546993531 | 0.036205076 | 0.55300308 | 4.493738 | 0.042203187 | 0.939155 |
| **Run 2** | 6.562428367 | 0.003466676 | 0.052826118 | 4.529814 | 0.032346575 | 0.714082 |
| **Run 3** | 6.566442718 | 0.004889086 | 0.074455631 | 4.550737 | 0.025918753 | 0.569551 |
| **Between Run Variation** | | | | | | |
|  | **Log6.6 Control** | | | **Log4.6 Control** | | |
|  | **Mean** | **St. Dev.** | **%CV** | **Mean** | **St. Dev.** | **%CV** |
|  | 6.558621539 | 0.020390185 | 0.310891318 | 4.524763 | 0.03870786 | 0.855467 |
